# CARDS, a Novel Prognostic Index for Risk Stratification and In-Hospital Monitoring

**DOI:** 10.3390/jcm13071961

**Published:** 2024-03-28

**Authors:** Siyu Liang, Qing Chang, Yuelun Zhang, Hanze Du, Huijuan Zhu, Shi Chen, Hui Pan

**Affiliations:** 1 Key Laboratory of Endocrinology of National Health Commission, Translation Medicine Centre, Department of Endocrinology, Peking Union Medical College Hospital, Peking Union Medical College, Chinese Academy of Medical Sciences (PUMCH, CAMS & PUMC), Beijing 100730, China; liangsy14@163.com (S.L.); duhanze@pumch.cn (H.D.); zhuhj@pumch.cn (H.Z.); 2 Medical Affairs, PUMCH, CAMS & PUMC, Beijing 100730, China; changq@pumch.cn; 3 Central Research Laboratory, PUMCH, CAMS & PUMC, Beijing 100730, China; zhangyuelun@pumch.cn

**Keywords:** sodium fluctuation, in-hospital mortality, hypernatremia, hyponatremia, monitoring

## Abstract

**Background**: Sodium fluctuation is independently associated with clinical deterioration. We developed and validated a prognostic index based on sodium fluctuation for risk stratification and in-hospital monitoring. **Methods**: This study included 33,323 adult patients hospitalized at a tertiary care hospital in 2014. The first 28,279 hospitalizations were analyzed to develop the model and then the validity of the model was tested using data from 5044 subsequent hospitalizations. We predict in-hospital mortality using age, comorbidity, range of sodium fluctuation, and duration of sodium fluctuation, abbreviated as CARDS. **Results**: In-hospital mortality was similar in the derivation (0.6%) and validation (0.4%) cohorts. In the derivation cohort, four independent risk factors for mortality were identified using logistic regression: age (66–75, 2 points; >75, 3 points); Charlson comorbidity index (>2, 5 points); range of sodium fluctuation (7–10, 4 points; >10, 10 points); and duration of fluctuation (≤3, 3 points). The AUC was 0.907 (95% CI: 0.885–0.928) in the derivation cohort and 0.932 (95% CI: 0.895–0.970) in the validation cohort. In the derivation cohort, in-hospital mortality was 0.106% in the low-risk group (0–7 points), 1.076% in the intermediate-risk group (8–14 points), and 8.463% in the high-risk group (15–21 points). In the validation cohort, in-hospital mortality was 0.049% in the low-risk group, 1.064% in the intermediate-risk group, and 8.403% in the high-risk group. **Conclusions**: These results suggest that patients at low, intermediate, and high risk for in-hospital mortality may be identified by CARDS mainly based on sodium fluctuation.

## 1. Introduction

Hyponatremia and hypernatremia are the most common electrolyte disorders in hospitalized patients. The prevalence of hyponatremia is 15%–30%, while the prevalence of hypernatremia varies from 0.9% to 26% [1,2,3]. Hyponatremia or hypernatremia is independently associated with an increased risk of mortality in hospitalized patients [3,4] and is responsible for a significant burden on the healthcare system [5,6]. Previous studies have shown that both the severity and duration of hyponatremia or hypernatremia are associated with an increased risk of mortality in hospitalized patients [7,8]. If these patients at higher risk of death can be predicted, it is expected that they can be identified in order to intervene in advance, and even more resources and monitoring can be prepared in advance, such as transfer to the intensive care unit.

Prevention of clinical deterioration and adverse events in hospitalized patients is an important issue for quality management in clinical practice. A variety of models have been developed to monitor and warn of in-hospital mortality in hospitalized patients. However, few of these models use electrolytes as the main predictor. Dysnatremia has only been used to assist in the prediction of in-hospital mortality, such as in the MET calling criteria. In contrast, there are few models that have been validated as highly accurate using electrolytes as the main predictor. This may be because previous studies have used hyponatremia and hypernatremia, which are significantly outside the normal range, to predict the outcomes of hospitalized patients. In contrast, recent studies have found that sodium fluctuations within the normal range of serum sodium are associated with in-hospital mortality [9,10,11,12,13]. We therefore propose that the range of serum sodium variability (also known as serum sodium fluctuations) is a better predictor of in-hospital mortality than either hypernatremia or hyponatremia.

The aim of the present study was to develop and validate a practical and user-friendly prognostic index for risk stratification and monitoring in hospitalized patients with higher accuracy. As an observational database, these data reflect real-world management patterns and in-hospital clinical outcomes for hospitalized patients.

## 2. Materials and Methods

### 2.1. Study Population and Definition

This single-center retrospective cohort study included patients admitted to Peking Union Medical College Hospital (Beijing, China) between 1 January 2014 and 31 December 2014. Patients aged 18 years or older, with at least two serum sodium measurements and with normal serum sodium at admission, were included in this study. Patients without serum sodium measurement within 24 h after admission were excluded from the analysis. All of the admissions were examined for the patients with several hospital admissions. Predictors of in-hospital mortality were determined from an initial derivation cohort consisting of data from 1 January to 31 October 2014. The validity of the model was then independently assessed using data from the second validation cohort, consisting of the subsequent hospitalization episodes from 1 November to 31 December 2014.

### 2.2. Data Extraction and Outcome

The data were extracted from the Electronic Medical Records of Peking Union Medical College Hospital. These data included demographic information, principal diagnosis, in-hospital death, and laboratory examinations, including all measurements of serum sodium during hospitalization. The primary diagnoses were grouped based on the International Classification of Diseases, 10th Revision, and the calculated Charlson comorbidity index (CCI). The sodium fluctuation was defined as the process between the highest and lowest levels among all serum sodium measurements during hospitalization. The range and duration of sodium fluctuation were calculated for every patient. The potential risk factors were chosen based on clinical relevance and previously reported predictors of mortality. The outcome of interest was defined as in-hospital death. Information about vital status was obtained from medical records, achieving 100% follow-up. This study was approved by the Clinical Research Ethics Committee of Peking Union Medical College Hospital (approval number: S-k1272, approval date: 9 October 2020).

### 2.3. Model Development

Three risk factors, including age, CCI, and range of sodium fluctuation, were identified according to our previous study [14]. The duration of fluctuation was introduced to the model as another independent risk factor to illustrate the rate at which sodium disorders develop. We measured the bivariable relationship between each risk factor and mortality in the derivation cohort using logistic regression models containing only the risk factor of interest. We then entered all risk factors associated with mortality into a multivariable logistic regression model to select the final set of risk factors. All variables included in the models were tested for collinearity, and odds ratios (ORs) with 95% confidence intervals (CIs) were computed. We constructed a prognostic index in which we assigned points to each risk factor by dividing each b coefficient in the final model by the lowest b coefficient (excluding the intercept term) and rounding to the nearest integer [15]. A risk score was assigned to each subject by adding up the points for each risk factor present. A panel discussion decided to divide all subjects into approximate tertiles based on their risk scores.

### 2.4. Model Validation

The accuracy of the risk index was determined by calculating the area under the receiver operating characteristic (ROC) curve (AUC) in both the derivation and validation cohorts. The AUC reflects the ability of the prognostic index to distinguish between patients at high and low risk of death. The calibration curve was plotted to graphically evaluate the consistency between actual and predicted death in the validation cohort. The ability of the prognostic index to identify inpatients at low, intermediate, and high risk for mortality was tested in a validation cohort. The patients were classified into three risk groups based on the prognostic index. Mortality for these risk groups and the mortality relative risks (RRs) and 95% CIs between risk groups were determined, and these data were compared with those of the derivation cohort. The accuracy of the risk index was further determined by AUC in subgroup analysis. The hyponatremia subgroup included all patients who developed hyponatremia during hospitalization in the validation cohort. The hypernatremia subgroup included all patients who developed hypernatremia during the hospitalization validation cohort.

### 2.5. Statistics

The normality of the distribution of continuous variables was tested by a one-sample Kolmogorov–Smirnov test. Continuous variables with normal distribution were presented as mean (standard deviation (SD)); non-normal variables were reported as median (interquartile range (IQR)). Means of 2 continuous normally distributed variables were compared by independent samples using Student’s *t*-test. Mann–Whitney U test was used to compare the means of 2 groups of variables not normally distributed. The frequencies of categorical variables were compared using Pearson χ^2^ or Fisher’s exact test when appropriate. A value of *p* < 0.05 was considered significant. All analyses were conducted with R (version 4.0.2, R Foundation for Statistical Computing, Vienna, Austria, 2020, https://www.R-project.org/ (accessed on 1 November 2020)).

## 3. Results

### 3.1. Patient Characteristics

**Figure 1:**
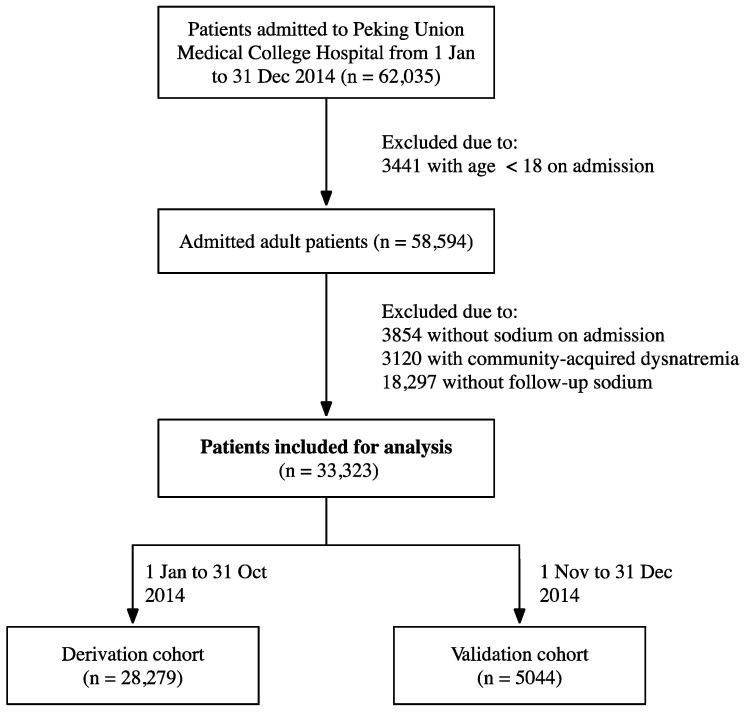
Study flow diagram of inclusion and exclusion criteria.

The process of inclusion and exclusion is shown in Figure 1. The baseline characteristics and main outcomes of the 28,279 hospitalization episodes used to develop the model (derivation cohort) and the 5044 hospitalization episodes used to test the model (validation cohort) are shown in Table 1. Fifty-two percent of patients in the derivation cohort were women. The mean (SD) age was 53.18 (15.36) years. The median CCI was 2. The mean range of sodium fluctuation was 5.92 mmol/L with a median duration of 9 days. The median length of stay was 8 days. One hundred seventy patients (0.6%) died during hospitalization. Fifty-two percent of patients in the validation cohort were women. The mean (SD) age was 53.11 (15.08) years. The median CCI was 2. The mean range of sodium fluctuation was 5.31 mmol/L with a median duration of 8 days. The average length of stay was 5 days. In total, 21 patients (0.4%) died during hospitalization. The in-hospital mortality was similar between the two cohorts (0.6% vs. 0.4%, *p* = 0.128).

### 3.2. Logistic Regression

Risk factors associated with in-hospital mortality in the bivariable analyses included the range and duration of sodium fluctuation, as well as the age and CCI (Table 2). All of these four risk factors were independently associated with mortality in the multivariable analysis (Table 2 and Table S1).

### 3.3. Risk Stratification with CARDS

This prognostic index was named CARDS based on the acronym of comorbidity, age, range, and duration of sodium fluctuation. The points assigned to the final four risk factors in the scoring system are listed in Table 3. A risk score was calculated for each patient by adding the points of each risk factor that was present. For example, an 80-year-old patient (3 points) admitted with a CCI of 3 (5 points) and a fluctuation in serum sodium by 10 mmol/L (4 points) within 3 days (3 points) would have a risk score of 15 points. Risk scores in the derivation cohort ranged from 0 to 21 points, with a mean (SD) score of 5.38 (4.71).

Patients were divided by risk scores into three risk groups, i.e., low- (0–7 points), intermediate- (8–14 points), and high-risk (15–21 points) groups. The clinical characteristics of patients in these three risk groups are summarized in Table S2. The RR of mortality between the intermediate- and low-risk groups was 10.24 (95% CI: 6.42–16.91). The RR of mortality between the high- and low-risk groups was 87.04 (95% CI: 55.71–141.59). Significant differences were detected between all risk groups (Table 4).

**Figure 2:**
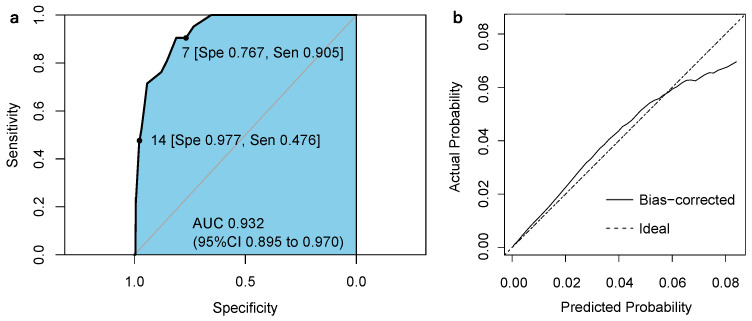
Receiver operating characteristics curve (**a**) and calibration curve (**b**) of the risk index for predicting in-hospital mortality in the validation cohort.

In-hospital mortality ranged from 0.106% (23/21,676) in the low-risk group to 8.463% (87/1028) in the high-risk group in the derivation cohort and from 0.049% (2/4079) to 8.403% (10/119) in the validation cohort (Table 4). The discrimination of our scoring system performed well in the validation cohort with an AUC of 0.932 (95% CI: 0.895–0.970), as shown in Figure 2a. The calibration plot demonstrated robust predictive performance with a close agreement between observed and predicted mortality (Figure 2b). In the subgroup analysis, 4056 patients in the validation cohort had hyponatremia during hospitalization. CARDS predicted in-hospital death with an AUC of 0.940 (95% CI 0.891–0.990) in the hyponatremia subgroup. While in the hypernatremia subgroup, CARDS predicted mortality with an AUC of 0.931 (95% CI 0.882–0.979) in 4742 patients.

### 3.4. Simplified Risk Stratification with CARDS

To simplify the risk stratification with CARDS, we calculated all possible combinations of the four risk factors. According to the score range of each risk group, patients were divided as follows: low-risk group, patients with sodium fluctuation ≤6 with another risk factor, or patients with sodium fluctuation ≤10 and no other risk factor; intermediate-risk group, patients with sodium fluctuation ≤6 and two to three additional risk factors, patients with sodium fluctuation ranged from 7–10 and one to two further risk factors, or patients with sodium fluctuation >10 and one more risk factor; and high-risk group, patients with sodium fluctuation ranged from 7–10 and all three other risk factors, or patients with sodium fluctuation >10 and two to three additional risk factors (Table 5).

## 4. Discussion

We have developed a prognostic index that can be used as a simple bedside risk-scoring system to help stratify hospitalized patients into high-, intermediate-, and low-risk groups for mortality. This index includes risk factors from four risk factors that could be abbreviated as CARDS: comorbidity, age, range, and duration of sodium fluctuation. This finding is consistent with the clinical scenario that in-hospital mortality is associated with sodium fluctuations [14]. Our index highlights the importance of considering sodium fluctuations when assessing prognosis in hospitalized patients.

Dysnatremia is the most common electrolyte disturbance in hospitalized patients [1,3]. Several studies have shown that hypernatremia (lowest sodium level below 135 mmol/L) and hyponatremia (highest sodium level above 145 mmol/L) are independently associated with increased mortality [1,3,4,16], particularly in patients with malignant tumors, central nervous system disorders, pulmonary disease, chronic kidney disease, HIV, heart failure or liver cirrhosis [3,17,18,19,20,21,22,23,24]. Recent studies have shown that sodium fluctuations are associated with in-hospital mortality, even within the normal range of serum sodium. In addition, hyponatremia and hypernatremia often occur in the same patient over a short time, called mixed dysnatremia. The prevalence of mixed dysnatremia was approximately 0.3% in hospitalized patients [25]. Mixed dysnatremia reflects rapid changes in serum sodium levels over a short period. These abnormalities can cause severe, permanent, or even fatal brain damage, whether the serum sodium level changes in the direction of lower or higher. In a study of 46,000 hospitalized patients, simple hyponatremia (HR 3.11, 95% CI 2.53–3.84), simple hypernatremia (HR 5.12, 95% CI 3.94–6.65), and mixed dysnatremia (HR 4.94, 95% CI 3.08–7.92) all led to an increased risk of in-hospital mortality [9]. Mixed dysnatremia may additionally increase the risk of in-hospital mortality [9]. When applying a serum sodium-related indicator to predict in-hospital death, we believe an indicator that can cover both hypernatremia and hyponatremia should be used. Many previous models that applied serum sodium to predict in-hospital deaths were those that considered only hyponatremia and hypernatremia. These models are less effective in predicting in-hospital deaths than using a single indicator of serum sodium fluctuation. Serum sodium fluctuation can more essentially reflect the imbalance of osmotic homeostasis in patients and better reflect the prognosis of patients than hyponatremia and hypernatremia [14]. We propose that changes in serum sodium with a higher dimensional and more unified perspective in hospitalized patients be observed.

Sodium fluctuation is an independent prognostic factor related to in-hospital mortality [9,10,11,12,13]. In the previous study, we reported that each 1 mmol/L fluctuation in serum sodium was independently associated with increased in-hospital mortality (adjusted OR: 1.20, 95% CI: 1.18–1.22, *p* < 0.001) [14]. We demonstrated the demographic and clinical characteristics of the patients in derivation and validation cohorts by a range of sodium fluctuation (Table S3). Although patients with more significant sodium fluctuations also had higher age and CCI, after correcting for the age and CCI, serum sodium fluctuation was independently associated with in-hospital death. Our findings are consistent with other studies. Lombardi et al. [9] found that the risk of in-hospital mortality increased linearly with an HR of 2.34 (95% CI: 1.55–3.54, *p* < 0.001) for each quartile of sodium fluctuation. Thongprayoon et al. [10] reported that the OR for in-hospital and 1-year mortality increased with sodium fluctuations in a dose-dependent manner from 1.47 to 5.48 in 60,944 inpatients. Sakr et al. [11] reported an OR of 1.55 (95% CI: 1.04–2.31, *p* = 0.033) for the sodium fluctuation > 6 mmol/L in 10,923 ICU patients. Topjian et al. [12] found in-hospital mortality associated with sodium fluctuation with an OR of 1.38 (95% CI: 1.06–1.8, *p* = 0.016) per 3 mmol/L in 380 PICU patients. Marshall et al. [13] observed an increased risk of in-hospital mortality per 1 mmol/L change in sodium fluctuation (OR: 1.10, 95% CI: 1.08–1.12, *p* < 0.001) in 8600 ICU patients. Sufficient evidence shows sodium fluctuation is an independent risk factor of in-hospital mortality.

Our observations first affirm an association between the duration of sodium fluctuation and in-hospital mortality (adjusted OR: 0.98, 95% CI: 0.98–0.99, *p* < 0.001). Fluctuation in a short period of time indicates an acute change in serum sodium. Dysnatremias are classified as acute if occurs within 2 to 3 days [26,27]. It is worth noting that the severity of dysnatremia symptoms depends largely on how quickly the dysnatremia progresses [28,29]. Acute hypernatremia causes cerebral shrinkage and an increased risk of vascular rupture. Acute hyponatremia results in cerebral edema, consequent seizures, coma, and death. Chronic dysnatremia exerts protective mechanisms that allow the brain to adapt to changes in serum sodium and reduces the dysnatremia. Our study directly proves that the relationship between sodium fluctuation and mortality is time-dependent.

There are several plausible explanations for the increased mortality among patients with sodium fluctuations. For example, in patients with chronic kidney disease, electrolyte disturbances can exacerbate arrhythmias and lead to sudden cardiac death [30,31]; in patients with heart failure, an increase in intracellular Na+ concentration prevents Ca^2+^ removal preventing energy production, which in turn may contribute to disease progression by inducing cell death and maladaptive remodeling [32,33]; in ischemia–reperfusion injury, the sodium–hydrogen exchangers pump Na+ into the cell for proton export, leading to disruptions to ion homeostasis [34]; in patients with liver cirrhosis, hyponatremia can impair brain function and predispose to hepatic encephalopathy, and improper fluid replacement can cause osmotic demyelination [35]. Death is often accompanied by serum sodium derangement in patients with heart failure, myocardial infarction, liver cirrhosis, pulmonary hypertension, pulmonary embolism, and chronic kidney disease [36]. These diseases may have the potential to induce the neurohumoral response. The neurohumoral response restores the arteries fulling through the activation of renin–angiotensin and the increase in vasopressin secretion, mediates the reabsorption of electrolyte-free water in the kidney, and subsequently inclines hyponatremia [37]. Fluctuations in serum sodium represent the degree and rapidity of neurohumoral activation and therefore indicate the severity of the underlying disease [36].

We first propose a prognostic index based on sodium fluctuation to risk stratification and in-hospital monitoring. CARDS has a high accuracy in predicting in-hospital mortality with only four risk factors. The AUC was 0.932 (95% CI: 0.895–0.970) with CARDS in the validation cohort, while the AUC was 0.852 (0.818–0.885) with sodium fluctuation as a single parameter [14]. Deviation in water–electrolyte balance can be represented by the range of fluctuation and the speed (duration) of changes in serum sodium. Rapid or significant changes in serum sodium are more likely to be ones accompanied by clinical deterioration. For example, acute hyponatremia can cause cerebral edema, but chronic hyponatremia usually does not. Moreover, even mild, chronic hyponatremia can lead to cognitive impairment, falls, and fractures, which are significant risks for the elderly or patients with multiple comorbidities [38]. Therefore, these four parameters can be used as predictors of in-hospital death.

Preventing clinical deterioration and adverse events in hospitalized patients is an essential issue in providing high-quality care in clinical practice [39]. A variety of models have been developed to monitor and warn of mortality in hospitalized patients. However, few of these models use electrolytes as the main predictor. Previously reported predictors have mainly focused on the changes in vital signs such as temperature, systolic blood pressure, respiratory rate, pulse rate, urine output, and level of consciousness, which often occur after clinical deterioration [40,41]. Electrolyte changes may occur before clinical deterioration and may theoretically help to predict death. Currently, hypernatremia or hyponatremia is only used as an adjunct to vital signs in predicting in-hospital mortality, such as in the MET calling criteria [42,43]. Predicting in-hospital mortality with hypernatremia or hyponatremia only had relatively low sensitivity, ranging from 7.3% to 52.8% [44,45,46,47,48]. This may be because previous studies used hyponatremia and hypernatremia, which are significantly outside the normal range, to predict in-hospital mortality. However, even variations in serum sodium within the normal range have been associated with poor outcomes. Aggregate-weighted systems with multiple parameters demonstrated slightly better prognostic performance. Wang et al. [49] built an aggregate weighted system with 322,046 electronic records based on both electrolyte and acid-base disturbances with an AUC of 0.81 (sensitivity of 65.4% and specificity of 88.4%). However, aggregate-weighted systems could not discriminate parameters needed for clinical interventions, were heavily equipment dependent, and thus had limited use in clinical practice. Our risk-scoring system has higher accuracy and better interpretability than previous systems. Risk stratification and monitoring with sodium fluctuation is a feasible and convenient method for the general hospitalized populations.

Potential limitations of the current analysis must be acknowledged. This is a retrospective study in a tertiary care hospital, which has a higher proportion of critical illnesses and a longer average length of stay than the general hospitals. The external validity should be evaluated in further studies. The 30-day mortality or 1-year mortality of admitted patients could not be obtained from the electronic system, leading to the absence of long-term prognostic observation. Despite these limitations, our study first proposed a prognostic index based mainly on sodium fluctuation for risk stratification and monitoring of in-hospital patients in an internal validation.

## 5. Conclusions

In conclusion, this study highlights the critical role of sodium fluctuation in predicting in-hospital mortality and presents a practical and user-friendly prognostic index (CARDS) for risk stratification in hospitalized patients. The index is more accurate and easier to use at the bedside than complex models using more electrolyte parameters. It provides valuable insights for clinicians, hospital administrators, and researchers in the management and understanding of water–electrolyte imbalances and their impact on clinical outcomes.

Future research should focus on validating the prognostic index (CARDS) in more prominent and diverse patient populations to enhance its robustness and generalizability. The validity of early prediction of in-hospital mortality should also be evaluated in further studies. Exploring the potential mechanisms underlying the association between sodium fluctuations and mortality may provide novel therapeutic targets. Integrating real-time sodium monitoring and implementing targeted interventions based on CARDS predictions may improve patient outcomes and reduce the burden on healthcare systems. Moreover, investigation of other electrolyte imbalances and their interactions with sodium fluctuations could provide a comprehensive understanding of electrolyte disorders in hospitalized patients. Collaboration between clinicians, researchers, and technology developers is essential to translate these findings into practical clinical tools and interventions that will ultimately improve patient care and outcomes.

## Figures and Tables

**Table 1:** Demographic and clinical characteristics of patients in derivation and validation cohorts.

	Derivation Cohort (*n* = 28,279)	Validation Cohort (*n* = 5044)
Female, No. (%)	14,567 (51.5)	2627 (52.1)
Age, mean (SD), yr	53.2 (15.4)	53.1 (15.1)
CCI, median (IQR)	2 (1–2)	2 (1–2)
Sodium, mean (SD), mmol/L		
Admission level	140.05 (2.36)	139.85 (2.25)
Lowest level	136.63 (3.69)	136.69 (3.34)
Highest level	142.55 (2.94)	142.00 (2.65)
Range of fluctuation, mean (SD), mmol/L	5.92 (4.51)	5.31 (3.98)
Duration of fluctuation, median (IQR), d	9 (3–44)	8 (3–33)
Length of stay, median (IQR), d	8 (3–15)	5 (1–12)

Abbreviations: CCI, Charlson comorbidity index; IQR, interquartile range; SD, standard deviation.

**Table 2:** Risk factors associated with in-hospital mortality in derivation cohort with bivariable and multivariable analyses.

	Bivariable		Multivariable	
	OR (95% CI)	*p*	OR (95% CI)	*p*
Age, per year	1.031 (1.020–1.042)	<0.001	1.024 (1.013–1.035)	<0.001
CCI	1.136 (1.117–1.155)	<0.001	1.138 (1.116–1.160)	<0.001
Range of fluctuation, per 1 mmol/L	1.200 (1.181–1.219)	<0.001	1.222 (1.200–1.244)	<0.001
Duration of fluctuation, per day	0.996 (0.992–0.999)	0.014	0.987 (0.983–0.991)	<0.001

Abbreviation: CCI, Charlson comorbidity index.

**Table 3:** Risk-scoring system of in-hospital death. Green: low-risk group, yellow: intermediate-risk group, red: high-risk group.

Risk Factor	Categories	Points
Age, yr	≤65	0
	66–75	2
	>75	3
CCI	≤2	0
	>2	5
Range of fluctuation, mmol/L	≤6	0
	7–10	4
	>10	10
Duration of fluctuation, d	>3	0
	≤3	3
Total score	0–7	Low risk
	8–14	Intermediate risk
	15–21	High risk

Abbreviation: CCI, Charlson comorbidity index.

**Table 4:** In-hospital mortality by risk stratification in derivation and validation cohorts.

	Derivation Cohort		Validation Cohort	
	RR (95% CI)	*p*	RR (95%CI)	*p*
Intermediate risk vs. low risk	10.24 (6.42–16.91)	<0.001	21.92 (5.64–143.95)	<0.001
High risk vs. low risk	87.04 (55.71–141.59)	<0.001	187.02 (48.58–1226.27)	<0.001
	No. Died/No. at Risk (%)	95%CI	No. Died/No. at Risk (%)	95%CI
Low risk	23/21,676 (0.106)	0.069–0.162	2/4079 (0.049)	0.008–0.198
Intermediate risk	60/5575 (1.076)	0.829–1.393	9/846 (1.064)	0.520–2.086
High risk	87/1028 (8.463)	6.868–10.376	10/119 (8.403)	4.327–15.290
AUC	0.907 (0.885–0.928)		0.932 (0.895–0.970)	

Abbreviation: AUC, the area under the receiver operating characteristic curve, is used to report the overall risk score.

**Table 5:** Simplified risk-scoring system for predicting in-hospital mortality. Green: low-risk group, yellow: intermediate-risk group, red: high-risk group.

Risk Factors *	Fluctuation Range		
	≤6	7–10	>10
0	0 point	4 points	10 points
1	2–5 points	6–10 points	12–15 points
2	5–8 points	9–12 points	15–18 points
3	10–11 points	14–15 points	20–21 points

* Risk factors including age > 65, Charlson comorbidity index > 2, duration of sodium fluctuation ≤ 3.

## Data Availability

Restrictions apply to the availability of data generated or analyzed during this study. The corresponding author will, on request, detail the restrictions and any conditions under which access to some data may be provided.

## Supplementary Materials

The following supporting information can be downloaded at: https://www.mdpi.com/article/10.3390/jcm13071961/s1, Table S1. Details of the logistic regression used to develop the risk scoring system. Table S2. Demographic and clinical characteristics of patients in derivation and validation cohorts by risk stratification. Table S3. Demographic and clinical characteristics of patients in derivation and validation cohorts by range of sodium fluctuation.
