# Supplementary material for: CARDS, a Novel Prognostic Index for Risk Stratification and In-Hospital Monitoring
Source: J Clin Med. 2024 Mar 28;13(7):1961. doi: 10.3390/jcm13071961 (PMC11012846; doi:10.3390/jcm13071961)
Supplement: Supplementary file 1 [file jcm-13-01961-s001.zip › jcm-2795960-supplementary.pdf]

Supplemental data

Table of contents:

Supplemental Table S1

Supplemental Table S2

Supplemental Table S3

Table S1. Details of the logistic regression used to develop the risk scoring system.

	Coefficient (b)	Standard error	z-value	P
Age	0.006	0.005612	4.163	<0.001
CCI	0.129	0.009829	13.166	<0.001
Range of fluctuation	0.200	0.009114	21.995	<0.001
Duration of fluctuation	-0.013	0.002268	-5.677	<0.001

Abbreviation: CCI, Charlson comorbidity index.

Table S2. Demographic and clinical characteristics of patients in derivation and validation cohorts by risk stratification.

Risk factor	Categories	Low risk No. (%)	Intermediate risk No. (%)	High risk No. (%)
Derivation cohort				
No.		21676	5575	1028
Age, yr	≤65	17629 (81.3)	3731 (66.9)	627 (61.0)
	66-75	3183 (14.7)	1130 (20.3)	226 (22.0)
	＞75	864 (4.0)	714 (12.8)	175 (17.0)
CCI	≤2	19305 (89.1)	2648 (47.5)	58 (5.6)
	＞2	2371 (10.9)	2927 (52.5)	970 (94.4)
Range of fluctuation, mmol/L	≤6	16689 (77.0)	1487 (26.7)	0 (0.0)
	7-10	4987 (23.0)	1647 (29.5)	37 (3.6)
	＞10	0 (0.0)	2441 (43.8)	991 (96.4)
Duration of fluctuation, d	＞3	16514 (76.2)	3635 (65.2)	821 (79.9)
	≤3	5162 (23.8)	1940 (34.8)	207 (20.1)
Validation cohort				
No.		4079	846	119
Age, yr	≤65	3346 (82.0)	561 (66.3)	68 (57.1)
	66-75	557 (13.7)	156 (18.4)	25 (21.0)
	＞75	176 (4.3)	129 (15.2)	26 (21.8)
CCI	≤2	3685 (90.3)	387 (45.7)	10 (8.4)
	＞2	394 (9.7)	459 (54.3)	109 (91.6)
Range of fluctuation, mmol/L	≤6	3217 (78.9)	295 (34.9)	0 (0.0)
	7-10	862 (21.1)	203 (24.0)	7 (5.9)
	＞10	0 (0.0)	348 (41.1)	112 (94.1)
Duration of fluctuation, d	＞3	3000 (73.5)	518 (61.2)	94 (79.0)
	≤3	1079 (26.5)	328 (38.8)	25 (21.0)

Abbreviation: CCI, Charlson comorbidity index.

Table S3. Demographic and clinical characteristics of patients in derivation and validation cohorts by range of sodium fluctuation.

		Range of serum sodium fluctuation No. (%)		
	Categories	≤6	7-10	>10
Derivation cohort				
No.		18176	6671	3432
Age, mean (SD), yr		52.48 (15.33)	54.67 (14.97)	53.98 (16.03)
Age, yr	≤65	14380 (79.1)	5042 (75.6)	2565 (74.7)
	66-75	2744 (15.1)	1196 (17.9)	599 (17.5)
	> 75	1052 (5.8)	433 (6.5)	268 (7.8)
CCI, mean (SD)		2.17 (2.88)	2.65 (3.23)	2.95 (3.84)
CCI	≤2	14318 (78.8)	5194 (77.9)	2499 (72.8)
	> 2	3858 (21.2)	1477 (22.1)	933 (27.2)
Duration of fluctuation, d	> 3	12396 (68.2)	5545 (83.1)	3029 (88.3)
	≤3	5780 (31.8)	1126 (16.9)	403 (11.7)
Risk of mortality	Low	16689 (91.8)	4987 (74.8)	0 (0.0)
	Intermediate	1487 (8.2)	1647 (24.7)	2441 (71.1)
	High	0 (0.0)	37 (0.6)	991 (28.9)
Validation cohort				
No.		3512	1072	460
Age, mean (SD), yr		52.60 (15.12)	53.94 (14.77)	55.07 (15.27)
Age, yr	≤65	2802 (79.8)	818 (76.3)	355 (77.2)
	66-75	490 (14.0)	187 (17.4)	61 (13.3)
	> 75	220 (6.3)	67 (6.2)	44 (9.6)
CCI, mean (SD)		2.23 (3.05)	2.48 (3.32)	2.82 (4.17)
CCI	≤2	2823 (80.4)	901 (84.0)	358 (77.8)
	> 2	689 (19.6)	171 (16.0)	102 (22.2)
Duration of fluctuation, d	> 3	2279 (64.9)	914 (85.3)	419 (91.1)
	≤3	1233 (35.1)	158 (14.7)	41 (8.9)
Risk of mortality	Low	3217 (91.6)	862 (80.4)	0 (0.0)
	Intermediate	295 (8.4)	203 (18.9)	348 (75.7)
	High	0 (0.0)	7 (0.7)	112 (24.3)

Abbreviations: CCI, Charlson comorbidity index; SD, standard deviation.
